# Investigating radical yield variations in FLASH and conventional proton irradiation via microscopic Monte Carlo simulations

**DOI:** 10.1088/1361-6560/add07b

**Published:** 2025-05-12

**Authors:** Yuting Peng, Youfang Lai, Lingshu Yin, Yujie Chi, Heng Li, Xun Jia

**Affiliations:** 1 Department of Radiation Oncology and Molecular Radiation Sciences, Johns Hopkins University, Baltimore, MD 21287, United States of America; 2 Department of Physics, University of Texas at Arlington, Arlington, TX 76019, United States of America

**Keywords:** FLASH, chemical yield, proton irradiation, radiation therapy, Monte Carlo simulation

## Abstract

*Objective.* Ultra-high-dose rate (UHDR) FLASH radiation therapy has shown remarkable tissue sparing effects compared to that at conventional dose rates (CDR). Radical production modulated by dose rate is expected to be one of the factors triggering different radiobiological responses. This study investigates the impacts of dose rate on radical yields in UHDR FLASH and CDR proton irradiation via GPU-based microscopic Monte Carlo (MC) simulations. *Approach.* We considered a region of interest (ROI) irradiated by a proton beam produced with a synchrotron pulse structure. The number of protons entering into the ROI was estimated in UHDR and CDR conditions. We sampled protons entering the ROI with randomly distributed spatial and temporal positions. An in-house developed GPU-based microscopic MC simulation package was used to model radiation physics and chemical processes with a periodic boundary condition. The temporal evolution of the radical yields was computed for different radical types, which in this work are hydrated electron $e_\textrm{h}$, hydroxyl $ ^{\cdot}\mathrm{OH}$, hydrogen radical $\mathrm{H}^\cdot$ and hydrogen peroxide $\mathrm{H_2O_2}$. We also examined radical yields with different proton energies from 1 to 142.4 MeV. *Main results.* Under the UHDR FLASH conditions, radical production was altered as a result of the spatial and temporal overlap of radicals produced by different protons, causing a change in their interactions. For the case with 142.4 MeV protons after 50 micropulses, the chemical yield of $ ^{\cdot}\mathrm{OH}$ under the FLASH scheme was decreased by ∼14% compared with that under the CDR condition. The percentage of reduction increased with the number of micropulses and decreased with proton energy. *Significance.* We modeled microscopic phenomena of radiation physics and chemistry triggered by synchrotron proton irradiation under UHDR FLASH and CDR conditions. Our results provided insights into the underlying mechanisms responsible for the FLASH effect.

## 1. Introduction

Ultra-high-dose rate (UHDR) FLASH therapy has emerged as a promising radio-therapeutic form for cancer treatment (Esplen *et al* 2020). Unlike the conventional radiation therapy (RT), which administers radiation at a relatively low dose rate of ∼0.1 Gy s^−1^, FLASH dose rates often exceed 40 Gy s^−1^. This technique has received considerable attention because of the potential reduction in normal tissue toxicities, while still maintaining local tumor control. The concept of FLASH dates back to 1959 (Dewey and Boag 1959). This study showed that bacteria (*Serratia marcescens*) exhibited reduced radiosensitivity under ultra-high dose rates (10–20 $\mathrm{kilorads}$/2 $\mathrm{\mu s}$) compared to normal dose rates (1000 $\mathrm{rads\,min}^{-1}$). The interest in FLASH renewed in 2014, when Favaudon *et al* (2014) provided compelling evidence of the FLASH sparing effect in a mouse model. Since then, the FLASH effect has been demonstrated in various normal tissues, including brain, skin and gut (Loo *et al* 2017, Montay-Gruel *et al* 2017, 2018, Levy *et al* 2019, Simmons *et al* 2019), as well as in larger animal models such as mini-pigs and cats (Vozenin *et al* 2019). However, not all studies have reported favorable outcomes. For instance, Bell *et al* (2025) demonstrated significantly impaired survival and no apparent differences in intestinal histology after proton FLASH irradiation compared to conventional dose rate (CDR) irradiation. The authors suggested that the mean dose rate and the time structure might be two important factors affecting the outcome. These results clearly highlighted the challenge towards clinical translation of FLASH RT, and in particular, the need to understand its radiobiological mechanism.

Current hypotheses for the FLASH mechanism can be broadly categorized into two groups: chemical changes at the molecular level, such as oxygen depletion, and biological effects at the cellular level, including mitochondrial damage (Borghini *et al* 2024, Ma *et al* 2024). In either case, the UHDR must play a role in triggering the different responses from that of the CDR situation. The immediate consequence of the substantially increased dose rate is a higher density of radicals within a given time frame, leading to enhanced interactions among radicals, as well as those between radicals and solutes. These chemical processes may trigger various downstream effects, affecting biological responses. The effects of the modulated radiolysis can be divided into two categories (Wardman 2020)—the depletion of a solute by radical-solute reactions and reduction in radicals by radical–radical reactions. One hypothesis for the FLASH mechanism based on radical-solute reactions is the oxygen depletion, which suggests that under UHDR conditions reduced oxidative damage to biological macromolecules, for instance lipid and DNA, may occur due to a transient oxygen depletion. This hypothesis has been extensively investigated by simulations (Pratx and Kapp 2019, Boscolo *et al* 2021, Lai *et al* 2021, Zhu *et al* 2021, Rabeya *et al* 2025) and experiments (Cao *et al* 2021, Jansen *et al* 2021). While the reduction in oxygen concentration under UHDR conditions has been observed and may contribute to the FLASH effect, a general consensus is that complete oxygen depletion is unlikely to occur within the clinical dose range (Atkinson *et al* 2023). Another hypothesis based on radical–radical reactions is the so-called inter-track effect due to chemical reactions between different primary particle tracks (Derksen *et al* 2023). Since chemical species, particularly $ ^{\cdot}\mathrm{OH}$, can cause indirect DNA damages, which may account for over two thirds of the total DNA damages (Lampe *et al* 2018), the inter-track effect can alter DNA damages and hence affect biology outcomes. This work will focus on the inter-track effect, characterized by the yield of chemical species under FLASH conditions.

FLASH effect is dependent on multiple parameters such as dose rates, dose, time of irradiation and pulse structures (Vozenin *et al* 2022), making it challenging to fully address the interplay between these factors. Microscopic Monte Carlo (MC) simulation is one of the most appropriate methods to investigate the FLASH mechanism for its ability and flexibility to offer insight into track structures, radical formation, and DNA damages (Nikjoo *et al* 2016), with nanometer-scale spatial and nanosecond-scale temporal resolution. Several studies have explored inter-track effects under the FLASH condition with microscopic MC method, reporting radical reductions when comparing radical yields in UHDR with CDR conditions. Alanazi *et al* (2021), Derksen *et al* (2023) and Thompson *et al* (2023) investigated radical yields by simulating water radiolysis triggered by multiple tracks simultaneously, albeit with different source particles and oxygen concentrations. Alanazi *et al* (2021) used 300 MeV protons and Derksen *et al* (2023) used 60 eV electrons and protons of 10 MeV and 100 MeV. Both work reported substantial reductions ($\gt$50%) of hydroxyl for proton cases. While Thompson *et al* (2023) also used 100 MeV proton, their results showed no inter-track effect at an evaluation time point of 1 ns and a very small reduction of hydroxyl yield (around 8% for delivered dose of 8 Gy) at 1 $\mu\mathrm{s}$. Baikalov *et al* (2023) employed microscopic MC simulations using 1 MeV electron tracks to fit model parameters, but the study also considered simultaneous tracks. As pointed out by Koch (2021), the simulation of multiple protons at the same time in a small volume resulted in ill-defined dose rates and impractical beam currents under clinical settings. A more realistic approach was used by Ramos-Méndez *et al* (2020), who explicitly simulated protons within a single pulse with pulse width varied from 1 ns to 10 $\mathrm{\mu s}$. Later, building on the methodology in Ramos-Méndez *et al* (2020), D-Kondo *et al* (2023) specifically investigated the inter-track effect on $\mathrm{H_2O_2}$ yield, using a pulse model whose widths were sampled from a Gaussian distribution.

In this paper, we report our recent studies on the impact of dose rate on radical yields in proton UHDR FLASH irradiation by synchrotron, compared with the yields in CDR irradiation, via microscopic MC simulation. Proton beams are advantageous for FLASH study. Energy of clinical proton beams can go up to 250 MeV with Bragg peak depth over 30 cm, making it suitable to treat deeply seated tumors compared to clinical electron beams, which typically penetrate only a few centimeters. It also allows the ‘shoot-through’ design to treat tumors located at the entrance plateau region, potentially making the patient setup more robust against density variations (Kneepkens *et al* 2023). High-energy protons can be produced by either cyclotrons or synchrotrons, but their time structures differ significantly, which is a crucial factor influencing chemical yields. In this study, we explicitly modeled the real synchrotron beam time structure at our institution, where FLASH experiments were conducted (Yin *et al* 2024). An in-house developed GPU-based MC package gMicroMC was used to support large-scale computations in this study (Lai *et al* 2020, Tsai *et al* 2020). To our knowledge, this is the first study to realistically model the radical production and evolution process triggered by protons under the pulse structure of a real synchrotron beam in FLASH compared to CDR settings using microscopic MC simulations.

## 2. Methods

### 2.1. Pulse structure of proton irradiation

The proton therapy system referred in this study (Hitachi Probeat CR, Hitachi Ltd Tokyo, Japan) is a multi-room synchrotron system designed for proton pencil-beam scanning. The synchrotron system delivers proton beams on a spill-by-spill basis. In each spill, a linear accelerator accelerates a number of protons to 7 MeV, and injects them into the synchrotron acceleration ring. The protons are then accelerated to a user-specified energy and extracted for irradiation.

**Figure 1.**
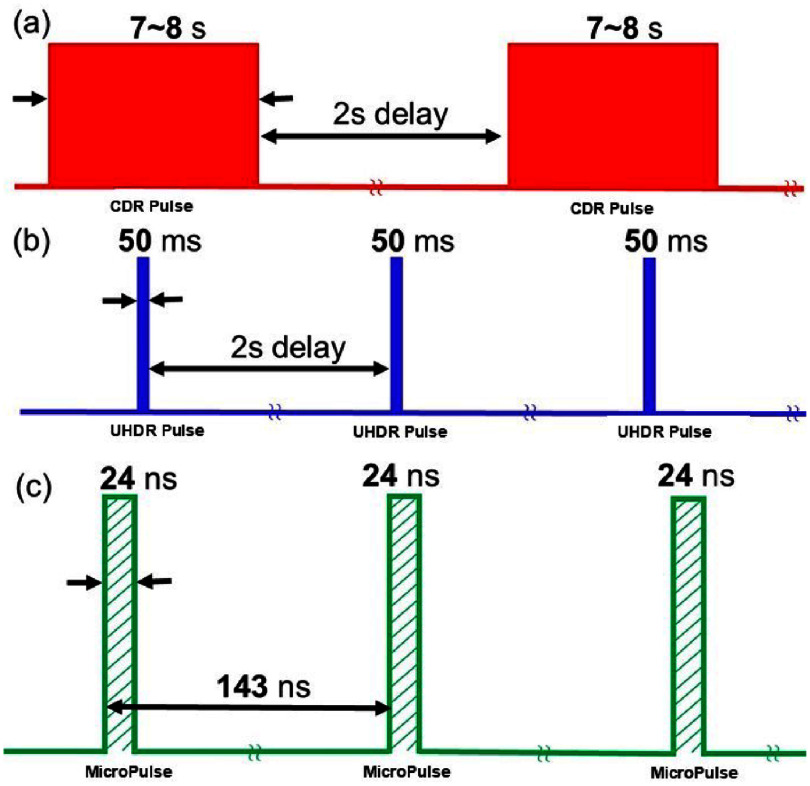
Schematic representation for the pulse structure of (a) CDR of maximal pulse width of 7–8 s and (b) UHDR proton beam irradiations. Note, the pulse widths vary with the doses to be used. (c) Micropulse structure for the synchrotron.

The pulse structures of the proton beam in CDR and UHDR modes are illustrated in figure 1. In the CDR mode (figure 1(a)), each pulse has a maximal width of 7–8 s to extract all protons from the synchrotron ring, with a 2 s delay between two adjacent pulses for replenishing protons to the ring. Note that the pulse width in an actual treatment varies depending on clinical scenarios.

In the UHDR FLASH mode (figure 1(b)), while the delay between adjacent pulses remains to be 2 s, the radiofrequency extraction power in the synchrotron system is increased to shorten the pulse length to $\Delta_\mathrm{UHDR} = 50~\mathrm{ms}$ (Hiramoto *et al* 2007, Yin *et al* 2024). In both the CDR and UHDR modes, during each pulse, the synchrotron delivers proton beams in a quasi-continuous mode with a micro-pulse structure of $\Delta_\mathrm{mp} = 24~\mathrm{ns}$ in pulse length and $T_\mathrm{mp} = 143~\mathrm{ns}$ in period (figure 1(c)).

In our proton system, we generated 142.4 MeV pulsed proton beams at ${\sim} 100$ nA beam current within the pulse to achieve an average UHDR dose rate of 726.3 Gy s^−1^ (Yin *et al* 2024). The maximum charge per spill was $Q = 4.96 \pm 0.10$ nC and it was extracted within one pulse for UHDR. The averaged proton rate within this pulse was hence

\begin{equation*} \bar{\Phi}_\mathrm{UHDR}^\mathrm{beam} = \frac{Q}{e\Delta_\mathrm{UHDR}} = 6.20\times 10^8\,\mathrm{ms}^{-1},\end{equation*}

where $e = 1.6\times 10^{-19}~\mathrm{C}$ is the charge of a proton. Therefore, given the micropulse width and period, the instantaneous proton rate within the micropulse was

\begin{equation*} \Phi_\mathrm{UHDR}^\mathrm{beam} = \bar{\Phi}_\mathrm{UHDR}^\mathrm{beam}\frac{T_\mathrm{mp}}{\Delta_\mathrm{mp}} = 3.69\times 10^9\,\mathrm{ms}^{-1}.\end{equation*}

Assuming a proton pencil beam spot with a Gaussian standard deviation *σ* = 3.0 mm (Yin *et al* 2024), the instantaneous flux (number of protons per unit time per unit area) at the pencil beam center, $\Psi_\mathrm{UHDR}$ was determined by

\begin{equation*} \Phi_\mathrm{UHDR}^\mathrm{beam} = \int\,\mathrm{d}x\mathrm{d}y\,\Psi_\mathrm{UHDR}^\mathrm{beam} \mathrm{e}^{-\frac{x^2+y^2}{2\sigma^2}}.\end{equation*}

Solving this equation yielded $\Psi_\mathrm{UHDR}^\mathrm{beam} = \Phi_\mathrm{UHDR}^\mathrm{beam}/2\pi\sigma^2 = 6.53 \times 10^9\,(\mathrm{cm^2\cdot ms})^{-1}$.

For the CDR scenario, the maximum charge in one spill is not necessarily extracted during one pulse and the actual protons per pulse vary with clinical cases. Thus, we estimate the instantaneous flux under CDR conditions from the dose rate reductions. The typical average dose rate during the irradiation is generally in the range of 0.1 to 1 Gy s^−1^ (Cao *et al* 2024, Tan *et al* 2024). Compared to the UHDR dose rate of 726.3 Gy s^−1^ (Yin *et al* 2024), a reduction in fluence by several orders of magnitude is expected for the CDR case. Assuming a conservative estimation of CDR dose rate of 1 Gy s^−1^, $\Phi_\mathrm{CDR}^\mathrm{beam}$ was calculated as $\Phi_\mathrm{UHDR}^\mathrm{beam} / 726.3 = 8.99 \times 10^6\,(\mathrm{cm^2\cdot ms})^{-1}$.

### 2.2. Simulation setup for UHDR scenario

#### 2.2.1. Region of interest (ROI) and Source modeling

The goal of this study was to investigate the impacts of dose rate on radical yields via microscopic MC simulations, and to compare the results between UHDR and CDR scenarios. In the UHDR case, we considered the experimental setting with a $2\times 2$ pattern with a spot separation of $a = 5~\mathrm{mm}$ (Yin *et al* 2024). The protons in the synchrotron ring were extracted within the 50 ms pulse and sequentially delivered to the four spots that formed a square pattern.

**Figure 2.**
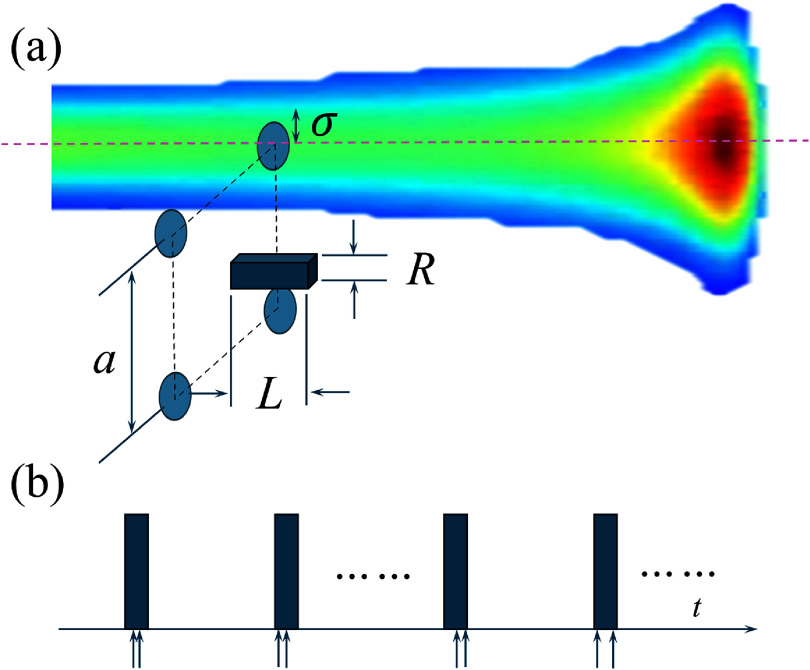
(a) Illustration of the simulation setup for the UHDR case. Objects are not drawn to scale. The color wash illustrates the 2D dose profile along the beam axis. The same setup was applied to CDR cases. (b) Illustration of proton injection time points for the UHDR case. Each arrow represents the moment a proton is injected into the ROI.

We considered a microscopic (of order of tens of micrometers) ROI of a rectangular box shape at the center of the square pattern, as illustrated in figure 2(a). The side of the box facing the proton beam was a square with a dimension of *R* = 50 $\mathrm{\mu m}$. For the UHDR FLASH irradiation duration $\Delta_\mathrm{UHDR} = $ 50 ms, the distance traveled by $ ^{\cdot}\mathrm{OH}$ can be estimated from the root-mean-square displacement of one-dimensional Brownian motion as $\lambda = \sqrt{2D\Delta_\mathrm{UHDR}} = 16.7~\mathrm{\mu m}$, where $D = 2.8\times 10^9~\mathrm{nm}^2\,\mathrm{s}^{-1}$ (Kreipl *et al* 2009) is the diffusion coefficient. The size of the ROI was hence large enough. As for the depth along the beam direction the ROI size was $L = 20~\mathrm{\mu m}$.

For the location at the center of the square beam pattern, the instantaneous proton flux within each micropulse is

\begin{equation*} \Psi_\mathrm{UHDR} = \Psi_\mathrm{UHDR}^\mathrm{beam}\mathrm{e}^{-\frac{\left(a/\sqrt{2}\right)^2}{2\sigma^2}} = 3.26\times 10^9\,\left(\mathrm{cm^2\cdot ms}\right)^{-1},\end{equation*}

where the term $a/\sqrt{2}$ is the distance between the square center to the spot center.

Given this proton flux $\Psi_\mathrm{UHDR}$, there are $n_\mathrm{proton} = \Psi_\mathrm{UHDR}R^2\Delta_\mathrm{mp} = 1.95$ protons entering into the ROI within each micropulse. We rounded this number to 2 for an integer number of protons per micropulse in our simulation.

Figure 2(b) shows the simulation scheme used to generate the incident protons with temporal randomness. For each micropulse, $n_\mathrm{proton} = 2$ protons are injected at random time points within its duration. The protons are assumed to be monoenergetic and to impinge perpendicularly at a random point on the front surface of the ROI.

#### 2.2.2. Microscopic MC simulations

Once the initial conditions of the protons were determined, we performed microscopic MC simulations using our in-house gMicroMC package to model the dynamic radiation physics and chemistry process triggered by the protons, focusing at computing the yields of chemical species *e*_h_, $ ^{\cdot}\mathrm{OH}$, $\mathrm{H}^\cdot$ and $\mathrm{H_2O_2}$, which can lead to oxidative damages to living cells and tissues, and comparing the yields between the CDR and UHDR scenarios. gMicroMC is a GPU-based MC simulation package for the simulations of the water radiolysis process and the computations of DNA damage (Lai *et al* 2020, Tsai *et al* 2020). It divides the simulation of the water radiolysis process into three stages: physical stage (${\unicode{x2A7D}}10^{-15}$ s), physicochemical stage ($10^{-15}-10^{-12}$ s) and chemical stage ($10^{-12}-10^{-6}$ s). During the physical stage, occurring on the femtosecond timescale, primary charged particles (protons in this study) and secondary electrons are transported, ionizing and exciting water molecules to generate reactive species. In the physicochemical stage, the de-excitation process of these water molecules is computed through predefined channels and probabilities, generating the initial distribution of radicals, which includes *e*_h_, $ ^{\cdot}\mathrm{OH}$, $\mathrm{H}_3\mathrm{O}^{+}$, $\mathrm{H}^\cdot$, $\mathrm{OH}^{-}$. These radicals then evolve through the thermalization process and reach thermal equilibrium around one picosecond after irradiation. After that, the simulation in the chemical stage models the diffusion of these radicals while considering mutual reactions among them in a step-by-step (SBS) simulation scheme. This stage is typically assumed to last for a few microseconds. Beyond that, local concentrations of radicals will be significantly attenuated and the cellular repair process will be at play (Bernal *et al* 2015).

In this work, water is used as the ROI material for both physics and chemical stages. No oxygen and scavengers were included in the chemical stage. A neutral environment (pH = 7) was used in the simulation. We modified the gMicroMC tool to continuously simulate the radiation chemistry process for multiple pulses in a SBS fashion, as well formulated in Karamitros *et al* (2014). The SBS method provides high temporal resolution and accurately preserves spatial and temporal correlations, making it well-suited for studying detailed reaction dynamics. In contrast, the more computationally efficient independent reaction time (IRT) method does not track the trajectories of individual chemical species but instead assumes independent reactive pairs (Clifford *et al* 1986, Plante and Devroye 2017). While IRT can be up to hundreds of times faster than SBS (Ramos-Méndez *et al* 2020), differences between the two methods may emerge in long-term behavior ($\gt$1 $\mathrm{\mu s}$) (Plante and Devroye 2017, Ramos-Méndez *et al* 2020). A detailed comparison is beyond the scope of this work. To maintain consistency with our previous work (Lai *et al* 2021), we opted to use the SBS method throughout our study.

Specifically, we modeled the irradiation process including a number of $n_\mathrm{mp}$ micropulses. After that, we continued the simulation for an additional $t_\mathrm{chem} = 1~\mathrm{\mu}$s for the chemical process to complete without additional proton injections. This setup leads to $t_\mathrm{end} = n_\mathrm{mp}T_\mathrm{mp}+t_\mathrm{chem}$. During this process, the step size was dynamically adjusted based on the configurations of the radicals (Tian *et al* 2017). At each time step, the radiation chemistry process was advanced to model the process of diffusion of radicals and mutual reactions among them. Once there was a new proton injection, we paused the simulation of the chemical process, completed the simulation of the primary proton’s physical and physicochemical stages, which was considered to happen instantaneously as compared to the relatively long chemical stage, and then continued the simulation of the chemical process of the entire ROI with the newly generated radicals from the injected proton. This process continued until the pre-defined simulation time $t_\mathrm{end}$ was reached.

#### 2.2.3. Boundary condition

Due to the diffusion process, the equilibrium of radicals close to the ROI boundary may be a concern. If a free boundary condition was used, the radicals generated within the ROI would diffuse outside. The lack of influx of radicals would lead to an underestimation of radical density and unrealistic modeling of the radiation chemical process. The problem was expected to become more severe in conditions with rapid radical productions, e.g. under the UHDR condition. To mitigate this problem, a periodic boundary condition along the six boundaries of the selected ROI was employed. Specifically, during the simulation, any radicals diffusing outside one side of the ROI were assumed to immediately re-enter into the ROI through the opposite side of the ROI.

#### 2.2.4. Calculation of chemical yields

During the simulation process, we recorded the total deposited energy *E* of the injected protons to the ROI at the end of the simulation $t_\mathrm{end}$. Meanwhile, we recorded the instantaneous radical numbers *R*(*t*) as a function of time *t*, including *e*_h_, $ ^{\cdot}\mathrm{OH}$, $\mathrm{H}^\cdot$ and $\mathrm{H_2O_2}$. The time step size of recording the data was 2 ps. We then computed the value of the chemical yield $G(t) = \frac{N(t)}{E} \times 100$, where *N*(*t*) is the number of radicals. Its unit is $\mathrm{molecules}/100\ \mathrm{eV}$ throughout this paper. Because of the randomness nature of MC simulations, radical yield values vary for one simulation to another even with the same simulation settings. Therefore, we repeated each simulation multiple times and reported the mean values. Each simulation was repeated 100 times except for simulations with 1 $\mathrm{MeV}$ proton, which were repeated 20 times. The resultant relative uncertainties of $ ^{\cdot}\mathrm{OH}$ yield for every simulation, defined as the ratio between the standard deviation and the mean value, were less than 3%.

### 2.3. Simulation setup for CDR scenario

The spot arrangement for CDR is the same as UHDR, as shown in figure 2(a). Following the same calculation, $\Psi_\mathrm{CDR}$ can be calculated from $\Psi_\mathrm{CDR}^\mathrm{beam}$ and its value was $4.49 \times 10^6\,(\mathrm{cm^2\cdot ms})^{-1}$. The computed number of protons per micropulse was $n_\mathrm{CDR} = R^2\Psi_\mathrm{CDR} \Delta_\mathrm{mp} = 0.0027$ with $R = 50 \mathrm{\mu m}$, which means that on average only one proton is injected into the ROI every 370 micropulses, or equivalently 52.9 $\mathrm{\mu s}$. This is a very long time compared to the life time of radicals and the chance of having radicals produced by subsequent protons overlapping with each other is low. Therefore, we modeled the CDR case as having independent protons injected into the ROI. For radical yield calculation, we only need to compute that of one proton. Similar to the UHDR case, we computed the radical yield *G*(*t*) for relevant species during $t_\mathrm{chem} = 1~\mathrm{\mu}$s after the injection of one proton.

### 2.4. Evaluations

We first presented the yields of chemical species, namely hydrated electron $e_\textrm{h}$, hydroxyl $ ^{\cdot}\mathrm{OH}$, hydrogen radical $\mathrm{H}^\cdot$ and hydrogen peroxide $\mathrm{H_2O_2}$, after the injection of one proton, which corresponds to the CDR case. This is a necessary step to confirm the calculation’s accuracy before moving onto more complicated UHDR cases. The proton energy was 30 MeV and the results were compared to experimental values. We also presented a simulation result for 142.4 MeV, which is the energy that achieved FLASH.

In addition to simulating the evolution of radical yield under the realistic experimental dose rate condition, we also studied the impact of a few other parameters. First, we investigated the impact of the number of micropulses. In the CDR case, due to the relatively large time interval between protons, which ensures that radicals from different proton tracks remain independent, the chemical yield is expected to be independent of the number of micropulses, and consequently, the total dose delivered. However, the situation differs in the UHDR case. To investigate this effect, we repeated the simulation for cases with different numbers of micropulses in the UHDR condition with 142.4 MeV protons but kept 2 protons per micropulse to maintain the same dose rate, and recorded the chemical yields at 1 $\mathrm{\mu}$s after the last micropulse.

It is also expected that the number of protons per micropulse plays a role in radical yields, because it again, affects the spatiotemporal overlap of radicals and hence the chemical reactions among them. This quantity is related to the dose rate in the experimental setting. The parameter of 2 protons per micropulse was derived from our experimental setting. Here to investigate the impact of this parameter, we varied the number of protons per micropulse in the range of 0.1 to 10 while maintaining a total of 100 protons, analogously to keeping the total dose constant. For the case with less than 1 proton per micropulse, we interpreted it as the probability of having a proton in the micropulse and hence sampled the proton accordingly. Specifically, we sampled a random number $\xi \in (0,1) $ for each micropulse. If *ξ* was smaller than the number of protons per micropulse, we sampled a new proton and included all the radicals it triggered in the chemical stage. Otherwise, we continued the chemical process without adding a new proton.

Finally, we investigated the impact of proton energy, or equivalently LET. Specifically, LET directly affects the spatial density and the amount of radicals produced at the end of the physical stage, and hence spatiotemporal evolution of the radicals. To study this effect, we varied proton energies from 142.4 $\mathrm{MeV}$ to 50, 10, and 1 $\mathrm{MeV}$, while maintaining $n_\mathrm{proton} = 2$ per micropulse. The simulation here may be interpreted as modeling the UHDR irradiation experiment at different depths in water, because proton flux can be approximately sustained as depth increases given the relatively low probability of proton-induced nuclear reactions. The chosen energies correspond to the entrance, plateau, pre-Bragg peak, and Bragg peak regions with LET values of 0.49, 1.19, 4.70, 40.29 keV *µ*m^−1^.

## 3. Results

### 3.1. Radical yields for CDR

**Figure 3.**
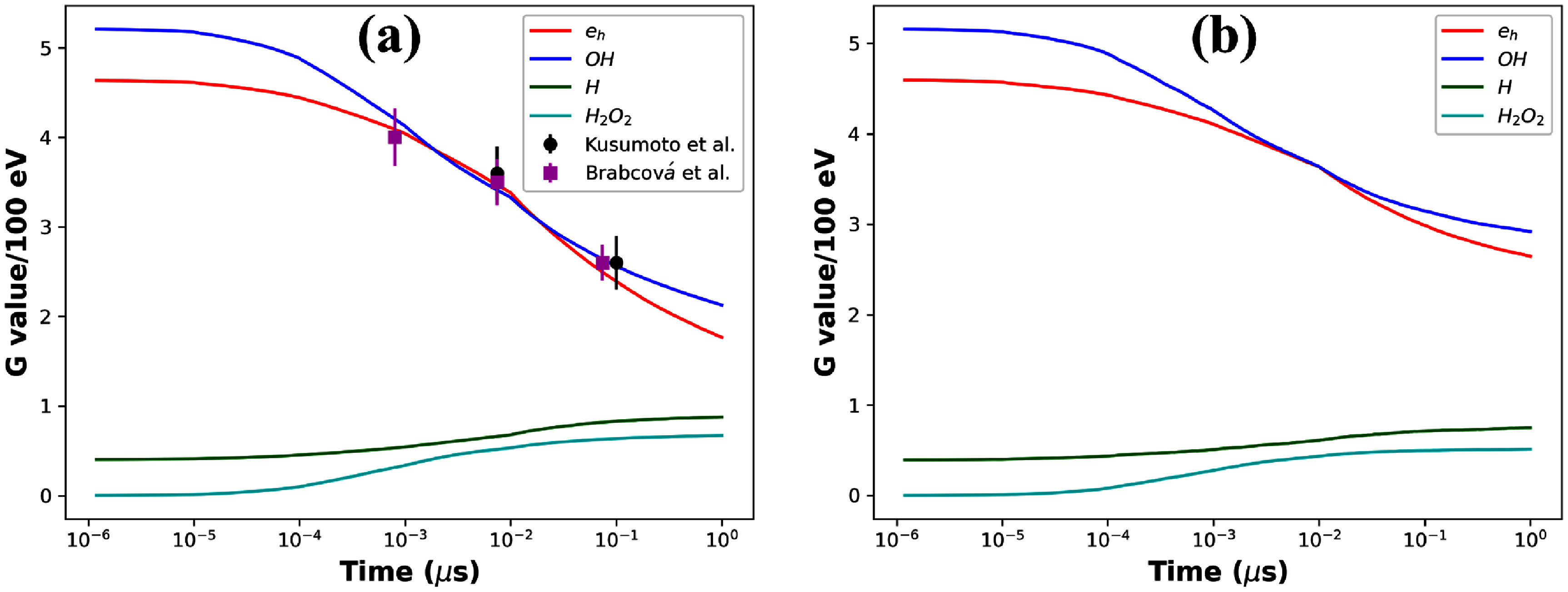
Radical yields as a function of time in the CDR case for a proton with energy (a) 30 MeV (b) 142.4 MeV. Experimental data of $ ^{\cdot}\mathrm{OH}$ at different time in (a) were extracted from Brabcová *et al* (2015), Kusumoto *et al* (2020).

Figure 3 shows the computed time evolution of yields for $e_\textrm{h}$, $ ^{\cdot}\mathrm{OH}$, $\mathrm{H}^\cdot$ and $\mathrm{H_2O_2}$ during the 1 $\mathrm{\mu}$s after the irradiation with 30 MeV and 142.4 MeV protons. Given that hydroxyl radical ($ ^{\cdot}\mathrm{OH}$) is highly reactive with biomolecules such as proteins and DNA, leading to indirect damages critical for cell killing, experiments have previously been carried out for 30 MeV irradiation (Brabcová *et al* 2015, Kusumoto *et al* 2020). We plotted the experiment result against our calculated ones in figure 3(a). The simulated $ ^{\cdot}\mathrm{OH}$ yield were found to align well with experimental values, with differences generally within 5%.

Figure 3(b) presents the radical yield evolution for a 142.4 MeV proton case. The result generally presented a similar form as the 30 MeV case, namely, the yields of $e_\textrm{h}$ and $ ^{\cdot}\mathrm{OH}$ drop while the yields of $\mathrm{H}^\cdot$ and $\mathrm{H_2O_2}$ increase with time, because of the rapid reactions among $e_\textrm{h}$, $ ^{\cdot}\mathrm{OH}$ and $H^+$. But the specific yield values at 1 $\mathrm{\mu s}$ differed. The G values in unit of $\mathrm{molecules}/100\ \mathrm{eV}$ of $e_\textrm{h}$, $ ^{\cdot}\mathrm{OH}$, $\mathrm{H}^\cdot$ and $\mathrm{H_2O_2}$ changed from 1.83, 2.19, 0.87 and 0.66 for 30 MeV protons to 2.75, 3.03, 0.74 and 0.49 for 142.4 MeV protons.

### 3.2. Dynamic evolution process of chemical yields

**Figure 4.**
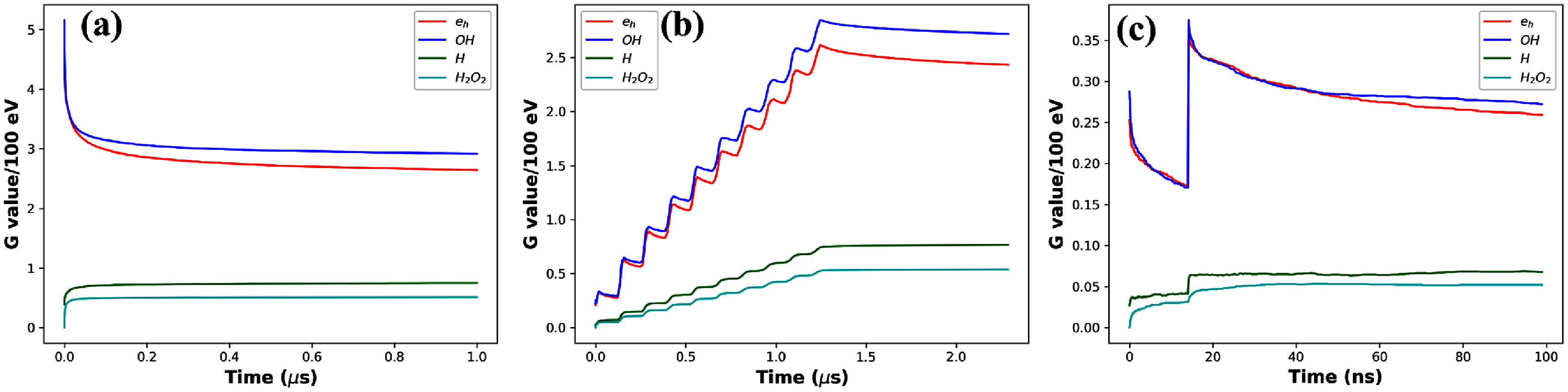
(a) Dynamic evolution process of chemical yields in the CDR case. (b) Dynamic evolution process of chemical yields in the UHDR case with 10 micropulses, and (c) zoom-in view of the first 100 ns for the UHDR case.

Figure 4 presents the dynamic evolution process of chemical yields in the CDR and UHDR FLASH cases with 142.4 MeV protons. Figure 4(a), the CDR case, is essentially the same result as figure 3(b) but plotted with a linear scale to facilitate the comparison with the UHDR case in figure 4(b). In the CDR case, the radical yields of $e_\textrm{h}$ and $ ^{\cdot}\mathrm{OH}$ first jumped to a high level after the proton injection, and then gradually decayed over the time, which eventually saturated to a stable level. The radical yields of $\mathrm{H}^\cdot$ and $\mathrm{H_2O_2}$ increased in this process and reached a steady state.

Figure 4(b) is a representative result for the UHDR case with $n_\mathrm{mp} = 10$ micropulses. There were 10 steps in the initial period of $n_\mathrm{mp}T_\mathrm{mp} = 1430$ ns, corresponding to the protons injected in the 10 micropulses. The zoom-in view of the first 100 ns of this process, as in figure 4(c), further presents the details during this short time interval. This interval was within the first proton micropulse. Two protons were injected, triggering the two spikes in $e_\textrm{h}$ and $ ^{\cdot}\mathrm{OH}$ yields, as well as the jumps in the $\mathrm{H}^\cdot$ and $\mathrm{H_2O_2}$ yields. Because of the relatively short time separations between protons, either within or between micropulses, as compared to the length of the radiation chemical stage, the chemical reaction process was altered by the rapid presence of protons as compared to that of the CDR case.

### 3.3. Radical yields in UHDR and CDR scenarios

To characterize the difference triggered by the dose rate between the CDR and UHDR scenarios, we computed the chemical yields 1 $\mathrm{\mu}$s after the irradiation. For the UHDR FLASH case, we computed that for 50 micropulses, as the radical yield started to saturate at this number of micropulses (see section 3.4.) Since the yield value is an intensity quantity, representing the number of radicals normalized by the energy delivered, the yield value in the CDR case is independent of the number of protons injected.

**Figure 5.**
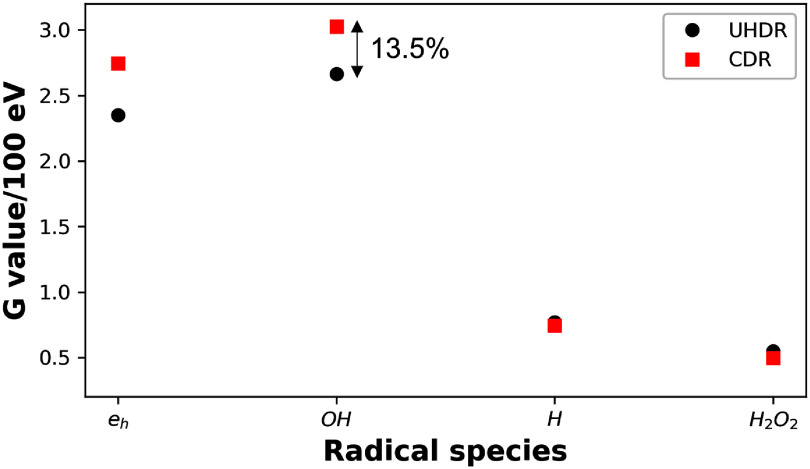
*G* values of different radicals for 142.4 MeV incident protons at the end of 50 micropulses for UHDR FLASH (black circle) and CDR (red square) conditions.

As shown in figure 5, in the UHDR case, after 1 $\mathrm{\mu}$s post the 50 micropulses the yield G values in unit of $\mathrm{molecules}/100\ \mathrm{eV}$ of radicals $e_\textrm{h}$, $ ^{\cdot}\mathrm{OH}$, $\mathrm{H}^\cdot$ and $\mathrm{H_2O_2}$ were 2.35, 2.65, 0.77 and 0.55, respectively. In contrast, these values were 2.75, 3.03, 0.74 and 0.49, respectively for the CDR setting. For the most biologically relevant one, $ ^{\cdot}\mathrm{OH}$, its chemical yield under the UHDR FLASH case was decreased by 13.5% compared with that under the CDR case. This decay is ascribed to the more rapid injection of protons in the FLASH case, which resulted in increased probability for radicals produced by protons both within and between micropulses to overlap with each other. The increased spatiotemporal overlap caused more intensive chemical reactions among radicals, leading to a reduction in $ ^{\cdot}\mathrm{OH}$ radicals.

### 3.4. Effect of number of micropulses and number of protons per micropulse

**Figure 6.**
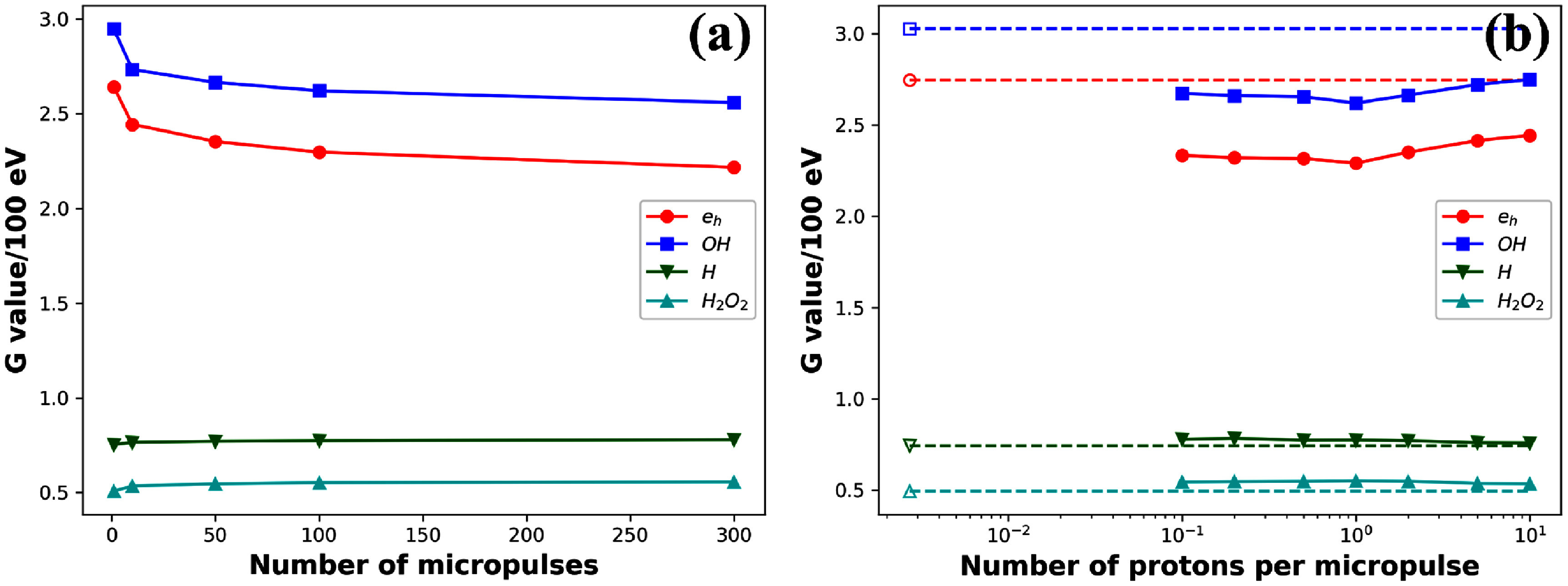
Dependence of radical yields on (a) number of micropulses for 2 protons per micropulse, and (b) number of protons per micropulse for 100 protons. Dash lines in (b) indicate levels of radicals in CDR scenario.

Figure 6(a) presents the dependence of radical yields on the number of micropulses. As the number of micropulses increased, the $ ^{\cdot}\mathrm{OH}$ yield gradually decreased. Approximately at 50 micropulses, the reduction started to saturate. At 300 micropulses, there were ∼16% reduction in $ ^{\cdot}\mathrm{OH}$ compared to the CDR case (2.55 vs 3.03 in unit of number per 100 eV).

Figure 6(b) presents the dependence of radical yields on the number of protons per micropulse. We also plotted in this figure the CDR case by putting its yields at $n_{\textrm{proton}} = 1/370$, according to the estimation in section 2.2.4. Comparing the CDR and UHDR FLASH cases, there was a clear distinction in radical yield values. Within the UHDR range, we observed a relatively weak dependence of radical yield on the number of protons per micropulse.

### 3.5. Effect of proton energy and LET

**Figure 7.**
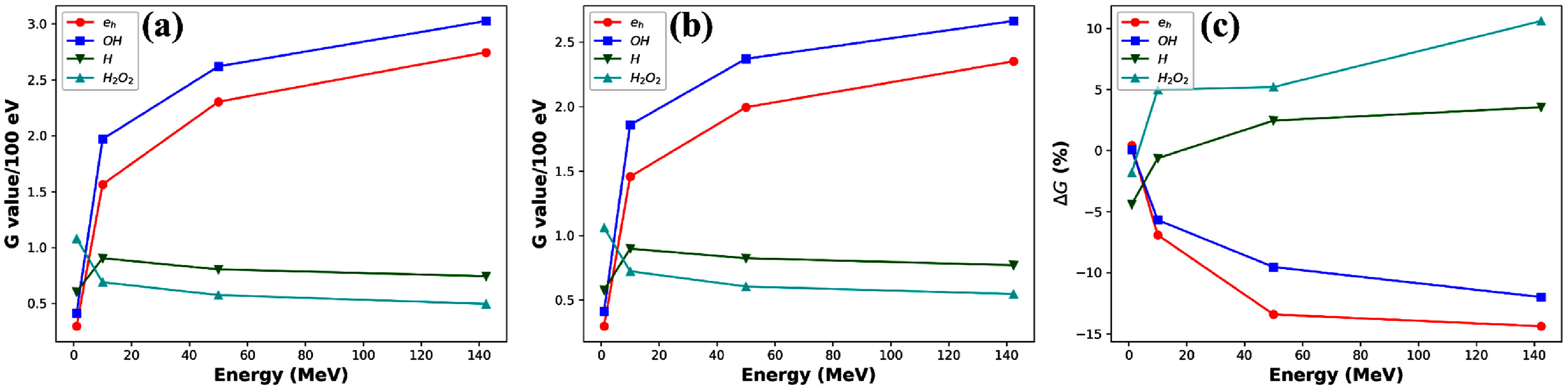
Dependence of radical yields on proton energy at (a) CDR and (b) UHDR FLASH conditions. (c) Relative changes of radical yields as a function of proton energy. Negative Δ*G* means a reduction of yield in the UHDR condition.

Figures 7(a) and (b) present the radical yield result under irradiation of protons with 142.4 MeV, 50 MeV, 10 MeV and 1 MeV for the CDR and UHDR FLASH scenarios at the proton fluence $\Psi_\mathrm{UHDR}$ for 50 micropulses, respectively. In both cases, the dependence of chemical yield on proton energy exhibited a similar trend. In particular, the $ ^{\cdot}\mathrm{OH}$ was decreased with reduced proton energy and increased LET.

Figure 7(c) shows the relative reduction of radical yields in the UHDR case compared to the CDR case, with a negative Δ*G* indicating a reduction in yield. For $ ^{\cdot}\mathrm{OH}$, it was observed that the radical yield reduction became less pronounced as the proton energy decreased.

## 4. Discussions

This study aimed to shed some light to the question regarding the impact of dose rate on the radical yields in proton beam FLASH therapy. Reactive species such as hydroxyl $ ^{\cdot}\mathrm{OH}$ and hydrated electron $e_\textrm{h}$ play a crucial role in inducing DNA strand and base damages (von Sonntag 2006), both of which contribute to DNA strand breaks and ultimately cell death. Radical-induced DNA damage has been reported to account for up to two-thirds of total DNA strand breaks (Lampe *et al* 2018). Additionally, these radicals drive lipid peroxidation and other molecular damage, leading to cellular dysfunction and triggering subsequent biological responses (Labarbe *et al* 2020). Given their critical role in radiation-induced damage, quantifying differences in radical yields between UHDR and CDR conditions is essential for understanding the mechanisms underlying the FLASH effect. For CDR, our simulation results agree well with the experimental values of $ ^{\cdot}\mathrm{OH}$ yield as shown in figure 3(a). Agreement improved at later post-irradiation time points, which was expected because the initial several nanoseconds involve highly heterogeneous radical distributions, making both experimental measurements and simulations more challenging. The key findings were that rapid proton injection in the UHDR FLASH condition altered the radiation chemistry process due to the spatially and temporally overlapped radicals produced by multiple protons. In particular, the results in figure 5 highlighted relatively significant reductions in chemical yields, specifically for $ ^{\cdot}\mathrm{OH}$ under FLASH conditions when compared with CDR scenario. An approximately 13.5% decrease in $ ^{\cdot}\mathrm{OH}$ yield was observed under the FLASH condition specific to the setting we modeled. This reduced yield can be attributed to the higher probability of radical interactions due to the dense spatiotemporal overlap in FLASH condition, leading to recombination and lower steady-state radical concentrations.

Furthermore, as the number of pulses increased in the simulation, the reduction of radicals became gradually more significant (figure 6(a)). The similar reduction trend with dose was also reported by Ramos-Méndez *et al* (2020), which can be ascribed to the shorter average distances between tracks with higher dose. The reduction level of $ ^{\cdot}\mathrm{OH}$ reached 16% at 300 micropulses. We did not continue the computations from hereon. This decision was made because of the consideration on the relevant time scale with respect to radical life time. In fact, our microscopic MC simulation did not consider radical life time, because we found in the literature that the life time data were associated with a relatively large uncertainty, and are strongly dependent on specific environment, e.g. cellular or water environment, which could be several nanoseconds and microseconds for hydroxyl (Von Sonntag 1987, Buxton *et al* 1988, Spinks and Woods 1990), respectively. Given that the 300 micropulses used in our simulation corresponded to 42.9 $\mathrm{\mu}$s, which was expected to be already longer than the life time of radicals, we anticipate our simulation should be valid for the situation with relevant radical life time considered.

Ideally, when evaluating the effect of dose rate, the same dose (i.e. number of protons) should be used in CDR as in UHDR cases. However, under the CDR conditions, the time interval between two primary protons is approximately 53 $\mathrm{\mu}$s. Keeping the same number of protons in CDR would yield a much larger number of time steps in simulation, leading to a prohibitive computational burden. Nonetheless, given that the typical lifetimes of hydrated electrons, hydroxyl radicals, and hydrogen radicals in pure water without scavengers are only a few microseconds (Von Sonntag 1987, Buxton *et al* 1988, Spinks and Woods 1990), which is much shorter than the inter-proton time interval, the water radiolysis processes initiated by individual protons can be reasonably considered independent. As a result, the radical yield from a single proton is representative for the CDR condition. Yet, this simplification may not be appropriate for long-lived species such as $\mathrm{H_2O_2}$, and should therefore be applied with caution.

We also would like to point out that, although random uncertainty can be reduced by repeated simulations, the results are still subject to systematic uncertainty. For instance, uncertainty in the MC simulation parameters inevitably affects the accuracy of the results. The uncertainty is associated with many factors, such as that of the physical interaction cross section data, that related to the chemical reaction data, as well as that in modeling the complex chemical reactions (Lai *et al* 2020). Nonetheless, we expect that qualitative behaviors discovered in this study, such as the reduction in steady-state radical yield of $ ^{\cdot}\mathrm{OH}$ under the UHDR condition, still hold. To understand the systematic uncertainty of the results, we scaled all physics cross sections by 20%, a typical experimental uncertainty reported in the literature (Muñoz *et al* 2008), and reran the simulations for both CDR and UHDR conditions with 50 micropulses. The *G* value for hydroxyl radicals (in units of number per 100 eV) changed from 3.03 to 2.91 for CDR, and from 2.65 to 2.58 for UHDR, resulting in an approximately 4% drop in absolute values for both conditions. The overall trend of radical reduction for UHDR compared to CDR was maintained, with the reduction ratio shifting slightly from 13.5% to 12.4%.

Proton energy was found to be a factor affecting radical yields, as observed in figure 7. While the UHDR FLASH condition maintained reduction in radical yield of $ ^{\cdot}\mathrm{OH}$ over the energy range, the effect becomes less pronounced at lower energies with higher LET values. There are two factors contributing to this effect. First, as LET increases with lower proton energies, production of radicals per unit length immediately after the physical stage was intensified, hence more intra-track reactions occurred subsequently, resulting in lower yields of radicals in CDR (figure 7(a)) and less room for FLASH effect in $ ^{\cdot}\mathrm{OH}$ radical reduction. Second, lower energy protons are more likely to produce low energy electrons with reduced travel length, making tracks less likely to overlap spatially. This result may suggest a LET dependence of the FLASH effect, which is also consistent with model-based analyses (Song *et al* 2023). A recent experiment using helium ions demonstrated that FLASH irradiation with 8 Gy dose in the entrance region (LET of 4.5 keV $\mathrm{\mu m}^{-1}$) resulted in significantly lower DNA damage compared to CDR irradiation. However, the same 8 Gy FLASH dose delivered at the middle of a spread-out Bragg peak (LET of 16 keV $\mathrm{\mu m}^{-1}$) showed a less pronounced effect (Tessonnier *et al* 2021).

Less hydroxyl reduction with increasing LET was also observed in another work (Ramos-Méndez *et al* 2020). However, their results showed hydroxyl reduction under UHDR only for LET $ < $2 keV $\mathrm{\mu m}^{-1}$ with pulse width $\gt$1 $\mathrm{\mu}$s and for LET $\lt$10 keV $\mathrm{\mu m}^{-1}$ with pulse width of 1 ns. In the contrary, our results showed hydroxyl reduction up to 40 keV $\mathrm{\mu m}^{-1}$, although the reduction becomes less pronounced with increasing LET. The reductions of $e_\textrm{h}$ and $ ^{\cdot}\mathrm{OH}$ under UHDR at 1 $\mathrm{\mu s}$ in our work are 15% and 13.5%, which is larger than the reported values of 6.1% and 8.6% for 1 $\mathrm{\mu s}$ pulse and 10.5% and 11.7% for 1 ns pulse (Ramos-Méndez *et al* 2020). The simulations with multiple protons simulated simultaneously provide substantially larger radical reduction than our results. Alanazi *et al* (2021) reported around 80% for both $e_\textrm{h}$ and $ ^{\cdot}\mathrm{OH}$. Derksen *et al* (2023) reported 54% reduction of $ ^{\cdot}\mathrm{OH}$. The difference is expected to be related with the ill-defined dose rate and dose in a small volume (Koch 2021), which proves the importance of correct consideration of the time structure.

Utilizing a GPU enables us to continue employing the SBS method for simulations, as a single GPU card (Nvidia Titan Xp GPU (1.58 GHz)) provides a ∼1000-fold acceleration over a single CPU core (Intel i7-6850 K CPU (3.6 GHz)) for a simulation of tracking 10^5^ molecules over 1 $\mu s$ (Lai *et al* 2021). Notably, according to our experience, this acceleration factor increases with the number of tracked chemical species, because better GPU occupancy can be achieved. For simulations with 50 micropulses, the number of chemical species ranged from approximately 10^5^ for 142.4 MeV protons to $3 \times 10^6$ for 1 MeV protons. Using our GPU-accelerated package on a moderate-performance NVIDIA T4 GPU, individual simulation times varied from 10 min to 2 h per run, requiring 20–40 h to complete a single irradiation condition with sufficient repetitions to reduce statistical uncertainty. Assuming an average simulation time of 30 GPU hours per proton energy, the equivalent CPU time required for four proton energies would exceed 5000 days, making it extremely challenging for traditional CPU-based computation.

One notable observation in the UHDR FLASH experiments is the reported reduction in $\mathrm{H_2O_2}$ yield (Montay-Gruel *et al* 2019, Blain *et al* 2022, Thomas *et al* 2024, Zhang *et al* 2024), which has been suggested as a possible explanation for the FLASH sparing effect (Montay-Gruel *et al* 2019). In contrast, our simulations, although consistent with other computational studies (Lai *et al* 2021, D-Kondo *et al* 2023, Derksen *et al* 2023), generally showed a slightly increased $\mathrm{H_2O_2}$ yield. This discrepancy warrants further discussion. The experimental measurement of $\mathrm{H_2O_2}$ yield often involves the addition of external chemicals, such as ammonium molybdate (Blain *et al* 2022), whereas our simulations were conducted in pure water. This introduces potential differences in pH, which is known to significantly influence $\mathrm{H_2O_2}$ production (Zhang *et al* 2024), or additional reactions with $ ^{\cdot}\mathrm{OH}$, the precursor of $\mathrm{H_2O_2}$, or $\mathrm{H_2O_2}$ itself. Furthermore, $\mathrm{H_2O_2}$ is a long-lived species, and its yield can be influenced by many chemical reactions with low reaction rates that occur over an extended time period. Some of these reactions, such as between $\mathrm{OH}^-$ and $\mathrm{H_2O_2}$, may be neglected in simulations but play a role when it comes to $\mathrm{H_2O_2}$. Future work will address the discrepancies by investigating the impact of these factors.

Our current simulation framework is inadequate for investigating tumor control probability preservation in UHDR FLASH, due to the omission of the tumor microenvironment in our modeling. This microenvironment, with its heterogeneous oxygenation, vascular structures, and immune interactions, profoundly influences radiation response, yet remains intractable for full micrometer-scale modeling. While reduced radical yields may lead to decreased biomolecular damages, their direct relevance to *in vitro* cell-based experiments remains uncertain due to the absence of a realistic cellular environment, including scavengers and oxygen (Wardman 2020, Koch 2021). The short lifetime of radicals within cells limits spatiotemporal track overlap, reducing inter-track effects. Conversely, radical scavenging introduces additional chemical species, further complicating reaction dynamics. This intricate interplay between radicals and intracellular solutes likely dictates radiation-induced damages. However, incorporating a realistic cellular environment into microscopic MC simulations is challenging due to the chemical complexity of intracellular species and the extended computational time required to model long-lived radicals associated with biomolecules such as lipids and DNA. While an ideal approach would include a comprehensive cellular model, a more practical and immediate alternative for benchmarking MC simulations is plasmid DNA experiments. These provide a controlled system where the chemical environment can be precisely manipulated. Notably, experiments have demonstrated the FLASH effect in reducing plasmid loss (Sforza *et al* 2024) in aqueous solutions with pH-stabilizing buffers, offering a well-defined comparison system. In future work, we plan to apply our simulation methods to such plasmid systems to assess whether modulated radiation chemistry influences DNA damage and structural integrity, paving the way for more complex cellular models.

## 5. Conclusion

In this paper, we investigated the impacts of dose rate on radical yields by simulating UHDR FLASH and CDR proton irradiations via GPU-based microscopic MC simulations. We estimated number of protons entering into a microscopic ROI based on dose rate for the irradiation of a proton beam produced with a synchrotron pulse structure. Using an in-house developed GPU-based microscopic MC simulation package, we modeled protons entering the ROI with randomly distributed spatiotemporal positions, and subsequently the radiation physics and chemical processes. Under the UHDR FLASH conditions, it was found that radical production was altered due to the spatial and temporal overlap of radicals produced by different protons and the mutual reactions among them. For the case with 142.4 MeV protons, at the end of 50 micropulses, the chemical yield of $ ^{\cdot}\mathrm{OH}$ under FLASH scheme was decreased by 14% compared with that under the CDR condition. The percentage of reduction increased with the number of micropulses and decreased with proton energy. This study provided a foundation for understanding inter-track effects under the synchrotron beam time structure. These findings offered important insights to the mechanisms of the FLASH effect and contributed to future modeling efforts aimed at optimizing UHDR radiotherapy.

## Data Availability

All data that support the findings of this study are included within the article (and any supplementary information files).
